# Neuromuscular Fatigue Profile of Prepubertal and Adult Female Handball Players

**DOI:** 10.3390/sports13070230

**Published:** 2025-07-11

**Authors:** Anastasia Papavasileiou, Eleni Bassa, Anthi Xenofondos, Panagiotis Meletakos, Konstantinos Noutsos, Dimitrios A. Patikas

**Affiliations:** 1 School of Physical Education and Sport Science at Serres, Faculty of Physical Education and Sports Sciences, Aristotle University of Thessaloniki, Ag. Ioannis, 62500 Serres, Greece; anastapt@auth.gr (A.P.); dpatikas@auth.gr (D.A.P.); 2 School of Health Sciences, Faculty of Life and Health Sciences, Frederick University, Nicosia 1036, Cyprus; a.xenofontos@frederick.ac.cy; 3 Department of Physical Education and Sport Science, National and Kapodistrian University of Athens, 17237 Athens, Greece; meletak@phed.uoa.gr (P.M.);

**Keywords:** child, female, recovery, athletic performance, handball, neuromuscular, electromyography, team sports, soleus, tibialis anterior

## Abstract

The investigation of the neuromuscular components of fatigue in team sports, especially in developmental ages, is limited. This study aimed to examine the neuromuscular fatigue and recovery patterns in prepubertal and adult female handball players, focusing on the soleus (SOL) and tibialis anterior (TA) muscles. Fifteen prepubertal (11.1 ± 0.9 years) and fourteen adult (22.0 ± 3.4 years) females performed a sustained isometric plantar flexion at 25% of maximal voluntary contraction (MVC) until exhaustion. The electromyographic (EMG) activity of the SOL and TA, torque, and central activation ratio (CAR) were recorded throughout the experiment. Endurance time was similar between groups (girls: 104 ± 93.5 s; women: 94.4 ± 30.2 s, *p* > 0.05), and both demonstrated progressive increases in muscle activation, without significant group differences for SOL and TA EMG (*p* > 0.05). Following fatigue, the torque and soleus (SOL) EMG activity decreased significantly compared to the pre-fatigue values in both groups (*p* < 0.001) and recovered (*p* > 0.05) in prepubertal and adult females within the first 3 and 6 min, respectively. The CAR remained unchanged over time, without significant differences observed between age groups (*p* > 0.05). These findings suggest that neuromuscular responses to fatigue are comparable between prepubertal and adult females, but recovery is significantly faster in prepubertal girls. Consequently, these findings underscore the need for age-specific recovery strategies in training programs, with tailored exercise-to-rest ratios to enhance performance and reduce fatigue during handball-specific activities.

## 1. Introduction

Handball is a high-intensity team sport that demands technical skill and coordination, characterized by frequent physical contact, sprinting, and rapid changes in direction [1]. Performance depends on technical and tactical ability, power, and sustained intermittent effort [2]. During a handball game, both male and female players operate at 82–87% of their maximum heart rate, utilizing both aerobic and anaerobic energy systems [3,4]. More specifically, female players cover a greater total distance per game (~4.7 km) than male players (~3.9 km), even though they cover a lower percentage of this distance with high-intensity running (2.5% compared to 7.9% for males) [5,6]. This suggests that female players are engaged more in continuous movements and less in high-intensity actions throughout the game, resulting in slightly lower overall intensity than males due to differences in game dynamics [2]. Given the limited literature available in this field, further research investigating the short-term physiological responses to exercise in female handball players, specifically in the context of fatigue, is warranted.

Fatigability arises from complex mechanisms of muscular and neural origins, where the interaction across different fatigue sites (peripheral and central) collectively impacts motor output and athletic performance [7]. During high-intensity fatigue protocols, female athletes tend to experience lower fatigue rates than their male counterparts [8,9,10]. This difference is explained by physiological and neuromuscular responses to exercise, which are influenced by hormonal fluctuations [11], body composition [12], and muscle fiber types distribution [13]. In handball, variations in fatigability may result in specific challenges for female players. A possible predominance of slow-twitch muscle fibers in females, while beneficial for endurance, might be less effective in producing force rapidly. This predominance might not provide the power required to prevent injuries during high-intensity tasks, involving explosive movements and frequent physical contact, as commonly observed during the game [14,15]. In line with this idea, several studies have reported notable differences in injury rates between male and female handball athletes, with females experiencing a higher incidence of injury (27.7 vs. 10.6 injuries/1000 h match) [16] and more severe injuries than males [17,18]. Moreover, during major international tournaments, 45% of injuries occur in the middle game (11–20 and 41–50 min), with the majority (54%) taking place in the second half [19], potentially as a result of the increased fatigability experienced by the players. These findings highlight the importance of investigating the fatigability of female athletes from a neuromuscular perspective in order to address and better understand these sex-specific disparities and inform possible modifications in training planning.

Despite the importance of this issue, there is a notable research gap concerning female athletes and the impact of age on muscle fatigue. Previous studies [20,21] have highlighted the influence of age and sex on skeletal muscle responses and fatigue during high-intensity intermittent exercise and protocols of maximum voluntary contraction, with children presenting a lower fatigue rate, attributed to differences in muscle composition, metabolic capabilities, and neuromuscular function. Regarding neural responses, only a few studies [22,23,24] have examined fatigue using electromyographic (EMG) data during isometric contractions in children. These studies highlight similar activation levels of the agonist and antagonist muscles between children and adults, with children recovering faster than adults [22,23,24]. However, relevant research has primarily focused on the male population [22,23,24,25,26], thereby limiting our understanding of the specific needs and challenges encountered by female athletes, including those participating in handball.

The persistently high risk of lower limb injuries among both adolescent and adult female athletes highlights the need for focused research on this population [18,27]. This risk is further supported by studies that emphasize the mechanical demands placed on the lower extremity joints during activities that involve running and jumping [28]. In such activities, improper fatigue management may increase the risk of injury due to the complex interplay between muscle function and joint mechanics [29,30]. Investigating fatigue, particularly in the soleus muscle, is critical, as it plays a pivotal role in maintaining stability and efficiency during locomotion. The soleus muscle is a major contributor to force generation during the stance phase of running and is optimized for prolonged work production due to its resistance to fatigue [29]. However, as fatigue develops, the muscle’s capacity to sustain this efficiency diminishes. As mentioned above, fatigue-induced declines in neuromuscular control may expose athletes to an increased risk of injury. Particularly, for the soleus muscle, fatigue may impact the stability of the lower limbs, which may lead to poor biomechanics and increased strain on other muscle groups and joints [31].

Given the predominance of slow-twitch (type I) fibers in the soleus, which are specialized for endurance and aerobic metabolism, it is particularly relevant to investigate fatigue using submaximal isometric contraction protocols. Unlike fatigue protocols with high intensity and short durations, submaximal fatigue protocols of longer duration demonstrate less pronounced differences between children and adults [32] and provide a suitable model for understanding fatigue over time, from the initial decline in force to complete exhaustion [33,34]. Therefore, submaximal fatigue protocols provide insights into the specific metabolic and neuromuscular processes that contribute to fatigue in the soleus muscle [33,34,35]. This is especially relevant in the young female population, which may exhibit distinct fatigue characteristics.

According to previous studies that emphasize the task- and sex-specific differences in muscle fatigue, there is a critical gap in our understanding of how fatigue manifests uniquely in females across various age groups [36,37]. Investigating the function of the neuromuscular system using techniques, such as EMG and nerve stimulation, provides a more comprehensive understanding of the neuromuscular response during sustained activities and during recovery. Considering the fact that the research on fatigue in young female athletes is limited, the purpose of this study was to assess the agonist and antagonist EMG activity before, during, and after a submaximal sustained isometric contraction in prepubertal and adult female handball players. Given that submaximal fatigue protocols show less subtle differences in fatigability between children and adults, our primary goal was to test whether this is also the case in female athletes of different ages, since training could potentially modulate metabolite production and neuromuscular responses differently among prepubertal and adult females during fatigue and recovery. Furthermore, examining the agonist and antagonist EMG activation may help us understand the underlying causes for the reduced force output due to fatigue. Through this investigation, the study seeks to investigate the relationship between age and neuromuscular adaptations to fatigue in handball athletes, providing valuable insights into the underlying mechanisms of muscle endurance.

## 2. Materials and Methods

### 2.1. Participants

Based on an a priori power analysis using G*Power software (v. 3.1.9.7, Franz Faul, University Kiel, Kiel, Germany) [38], a sample size of 28 participants was estimated as sufficient to detect an effect size of 0.25, with the statistical power and significance level (α) set at 0.80 and 0.05, respectively. Non-probabilistic convenience sampling was used to recruit prepubertal and adult female handball players from two local handball teams. Fourteen adult (mean ± SD age: 22.0 ± 3.4 years, body mass: 63.5 ± 5.5 kg, height: 168.6 ± 4.7 cm, training experience: 5.1 ± 1.6 years) and 15 prepubertal (age: 11.1 ± 0.9 years, body mass: 45.4 ± 7.2 kg, height: 152.3 ± 6.5 cm, training experience: 2.5 ± 0.8 years) females voluntarily participated in this study.

The participants were handball athletes who were free of any orthopedic injury or neuromuscular deficit. All eligible participants were able to complete the testing protocol without any complications. They were actively involved in competitive handball training during the in-season phase of their respective championships. The prepubertal females trained 4 to 5 h per week and competed at the regional youth level, typically participating in one official match each week. The adult females were engaged in 6 to 8 h of training per week and competed in the first (n = 9) or second (n = 5) division of the National championship, with one match per week during the assessment period. No participant had taken any nutritional supplements during the previous 6 months, and all participants were instructed to follow their usual dietary habits and restrict caffeine and alcohol consumption 24 h prior to the assessment.

Before the experimental procedure, all participants, as well as the parents or legal guardians of the children, were informed about the experimental protocol and gave their informed consent. All young females were classified as prepubescent (stage 1 or 2) according to the Tanner criteria [39]. The experiment was performed with the approval of the Aristotle University of Thessaloniki’s ethics committee, as outlined in the 6th Declaration of Helsinki. All measurements were performed at the Laboratory of Evaluation of Human Biological Performance of the Aristotle University of Thessaloniki.

### 2.2. Instrumentation

An isokinetic dynamometer (CYBEX Norm Cybex division of Lumex, Ronkonkoma, NY, USA) was used for the isometric torque measurements and the fatigue task. Prior to each test, the dynamometer was calibrated according to the manufacturer’s guidelines. Each participant was seated comfortably on the isokinetic dynamometer chair with the hip joint positioned at 90° of flexion, the knee in full extension (0°), and the ankle at 90°. Joint angles were confirmed using a handheld goniometer. The ankle joint was aligned so that the dynamometer’s axis of rotation corresponded to the anatomical axis of the ankle, which was approximated by the line connecting the medial and lateral malleoli. Velcro straps stabilized the torso and thigh on the chair and the foot on the dynamometer’s platform.

The EMG activity of the soleus and tibialis anterior muscles was recorded using bipolar surface electrodes (Ag-AgCl) 0.8 cm in diameter, with 1.2 cm inter-electrode distance. The conductive surfaces of the electrodes were covered with conductive paste and placed over the muscle belly according to the SENIAM recommendations [40]. Prior to electrode placement, the surface of the skin was shaved, abraded with sandpaper, and cleaned with alcohol wipes. The EMG signal was amplified (×1000 from 20 Hz to 3 kHz) and digitized (12-bit at a sampling rate of 4 kHz) with an A/D converter (Biopac MP150 Systems Inc., Goleta, CA, USA).

For the stimulation of the tibial nerve, a square pulse 1 ms in duration was delivered via surface electrodes using an electrical stimulator (S88, Grass Instruments, Quincy, MA, USA). The anode (3.5 × 5.5 cm) was placed below the patella, and the cathode (0.8 cm diameter) was positioned in the popliteal fossa. The optimal position was identified by making slight displacements of the cathode while stimulating at submaximal intensity and observing the magnitude of the M-wave and torque. The cathode was moved to a more distal and medial position if any sign of dorsiflexion was detected. Velcro straps were used to fix the cathode in place. The intensity was set at ×1.2 the intensity that produced an M-wave with a maximum peak-to-peak amplitude (supramaximal intensity).

### 2.3. Testing Procedures

The participants were familiarized with the testing procedures and the experimental protocol. Prior to the MVC assessments, all participants completed a standardized warm-up protocol, consisting of 5 min of treadmill walking at a self-selected pace, followed by dynamic stretching and mobility exercises targeting the lower limbs, including ankle circles and heel-to-toe swings. Subsequently, participants performed three submaximal isometric plantar flexion contractions, with progressively increasing intensity, based on their perceived maximal effort. Each contraction was held for 3–5 s, with a 30 s rest interval between trials. This gradual ramp-up protocol was designed to activate and prepare the muscle groups of interest and to allow the participants to become familiar with the testing environment. Five minutes after the warm-up, three 3–5 s isometric maximal voluntary contractions (MVCs) for the plantar flexors were performed, before the sustained isometric protocol, with 5 min rests after each trial. The participants were instructed to generate their maximum force as quickly as possible and to maintain the contraction for up to 5 s. Verbal encouragement to promote maximum effort was provided by the same investigator during all testing sessions. Additionally, real-time visual feedback of the torque output was displayed on a monitor placed in front of the participants to guide and reinforce maximum performance. During the plantarflexion MVC, the EMG signals of the soleus (agonist) and tibialis anterior (antagonist) muscles were recorded synchronously. The trial with the highest torque was used as a reference to set the contraction intensity for the fatigue protocol at 25% of MVC.

After the estimation of the maximal torque, each participant performed 3 isometric contractions at 10, 20, 30, 40, 50, and 60% of the MVC in random order while the torque and EMG were recorded. The duration of each trial was 2–3 s, the interval between the trials was set at 30–60 s, and visual feedback of the torque was shown on a screen, with a horizontal line set at the target torque.

During the fatigue protocol, the participants were requested to keep their torque output as close as possible to 25% of their MVC. The protocol was terminated at the endurance limit, which was defined as the time point when, despite maximal effort, torque could not be retained above 20% of MVC for more than 5 s. Verbal encouragement was given to all subjects, especially before the end of the protocol.

After the end of the fatigue protocol, maximum isometric contractions 3–5 s in duration were recorded immediately after and during the 3rd and 6th minutes after the end of the fatigue protocol (Post0, Post3, and Post6). During the MVC at Post0, Post3, and Post6, a single supramaximal stimulus was applied when torque reached a plateau.

### 2.4. Data Analysis

All recordings were visually inspected for integrity and quality and then further processed using custom Matlab scripts (Matlab R2022b; Mathworks, Inc., Natick, MA, USA).

To quantify the muscle activation in the soleus and tibialis anterior muscles, the EMG signals were filtered (using a 4th-order Butterworth band-pass filter at 10–500 Hz), and the root mean square (RMS), with a window of 40 ms, was calculated (SOL_RMS_ and TA_RMS_, respectively). The maximum torque and EMG were identified as the mean torque and RMS EMG, respectively, 0.2 s before and after the peak torque of the MVC trial in which the participant exhibited the best performance in terms of torque. For the torque and EMG during the sustained isometric contraction, the mean values were calculated over a period of 0.4 s, every 10 s. All RMS EMG values during the sustained isometric contraction were expressed as a percentage of the RMS calculated during the MVC trial. Furthermore, time was normalized to the endurance time for each subject and was expressed as a percentage. Each fatigue trial was divided into 5 segments of 20% of the endurance time (0–20, 20–40, 40–60, 60–80, and 80–100% of the endurance limit), and all recordings of each segment were averaged.

From the measurements of the EMG at different levels of voluntary contraction, the coefficients of the regression line (slope and intercept) were calculated using the RMS data as the independent variable and the percentage of torque produced as the dependent variable. These coefficients were used to estimate the percentage of plantar flexion torque (T_SOL_) based on the soleus RMS EMG during sustained contraction and using the coefficients of the EMG–torque relationship.

To estimate the central activation ratio (CAR) after fatigue, the mean torque 0.2 s before (T_before_) and the peak torque 0.3 s after (T_after_) the supramaximal stimulation were evaluated. The CAR was calculated using the following formula:

$$\mathrm{CAR}=\frac{T_{before}}{T_{after}}\times 100\%$$

### 2.5. Statistical Analysis

Means and standard deviations (SDs) for all dependent variables were calculated. Plantar flexion torque, SOL_RMS_, TA_RMS_, T_SOL_, and CAR were treated as dependent variables. A two-way repeated measure analysis of variance (ANOVA) was used to calculate the main effects for the GROUP (girls and women) and TIME (segments of endurance limit or measurements before fatigue and during recovery) factors, as well as their interaction. The normality of data distribution was assessed using the Shapiro–Wilk test, and the assumption of equality of variance–covariance was confirmed using Levene’s test. Differences between the individual subgroups were analyzed using a Tukey post hoc test. Furthermore, the effect size (Cohen’s d) of the statistically significant differences was also calculated, and the values were interpreted as negligible (d ≤ 0.2), small (0.2 < d ≤ 0.5), moderate (0.5 < d ≤ 0.8), or large (d > 0.8). The level of significance, α, was set at 0.05. The F-values and *p*-values of the main effects and the interaction are reported. All statistical tests were assessed using Statistica v.8.0 (Statsoft Inc., Tulsa, OK, USA) and R v.4.5.1 (R Development Core Team, Vienna, Austria).

## 3. Results

### 3.1. During Sustained Isometric Contraction

During the sustained isometric contraction, mean torque, expressed as a percentage of torque, was 25.2 ± 5.5% for girls and 26.6 ± 4.2% of the MVC for women, whereas the fatigue protocol lasted for an average of 104.0 ± 93.5 s in girls and 94.4 ± 30.2 s in women. For these parameters, there was no statistically significant difference between the groups.

**Figure 1:**
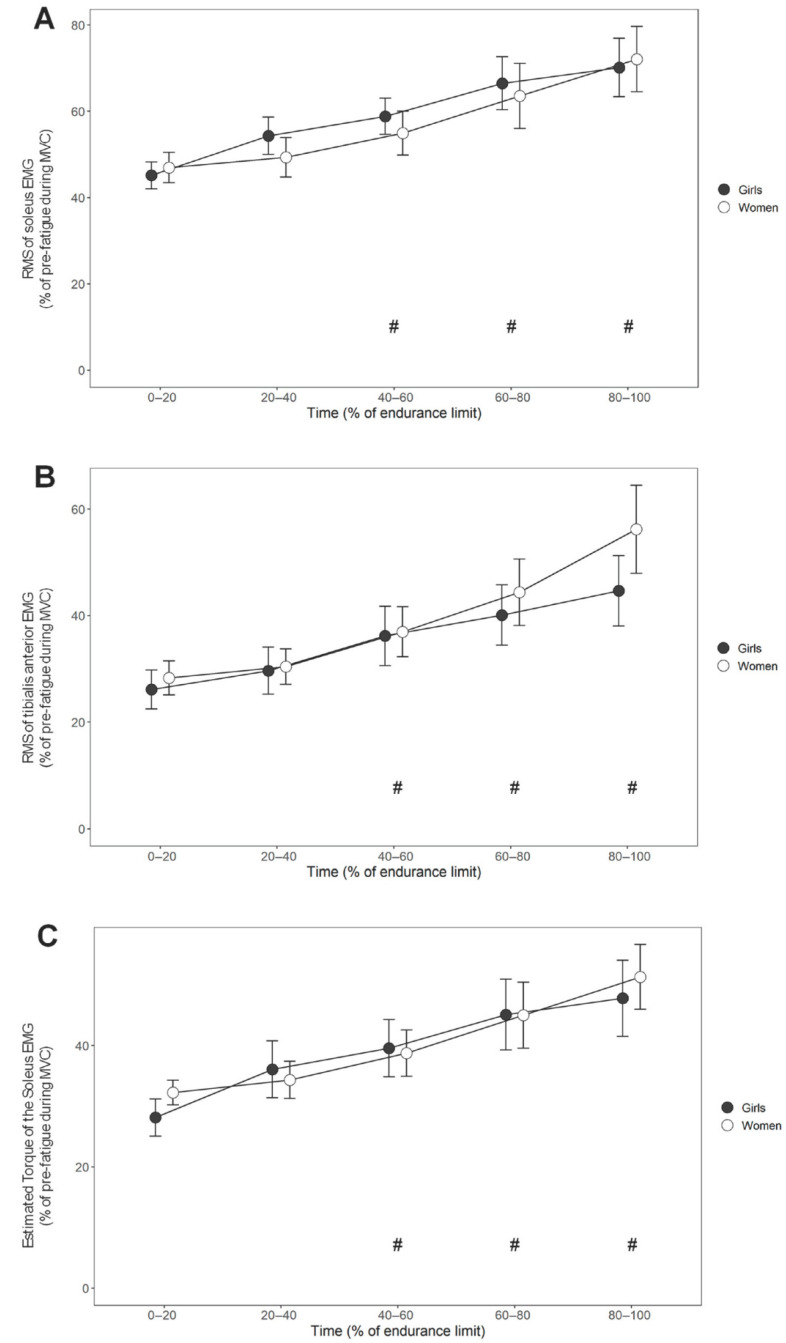
Group means and SDs of the soleus (**A**) and tibialis anterior (**B**) RMS EMG activity, along with the estimated torque based on the EMG/torque relationship before fatigue (**C**) across different time intervals (% of endurance limit). #: Significant time effect compared to 0–20 values.

SOL_RMS_ during the fatigue protocol (Figure 1A) revealed no differences in muscle activation between girls and women (F = 0.1, *p* = 0.813) and a significant time effect (F = 22.5, *p* < 0.001), demonstrating an increase in muscle activation during the protocol. SOL_RMS_ in both the girls and women increased progressively across the time intervals, with significant differences observed after 40% of the endurance time (40–60%: *p* = 0.004, d = 0.9 large; 60–80%: *p* ≤ 0.001, d = 0.9 large; 80–100%: *p* < 0.001, d = 1.1 large). No statistically significant GROUP × TIME interaction was observed (F = 0.6, *p* = 0.664), suggesting that both groups exhibited a similar increase in soleus muscle activation throughout the fatigue protocol.

TA_RMS_ (Figure 1B) demonstrated no significant differences between the groups (F = 0.3, *p* = 0.573), indicating that both girls and women exhibited similar muscle recruitment patterns throughout the fatigue protocol. Similarly to SOL_RMS_, a significant time effect was observed (F = 25.9, *p* < 0.001), with TA_RMS_ progressively increasing after 40% of the endurance time (40–60%: *p* = 0.004, d = 0.7 moderate; 60–80%: *p* ≤ 0.001, d = 1.0 large; 80–100%: *p* < 0.001, d = 1.1 large). This pattern was consistent in both groups, with no statistically significant GROUP × TIME interaction (F = 1.5, *p* = 0.208).

T_SOL_ during fatigue (Figure 1C) revealed no statistically significant GROUP effect (F < 0.1, *p* = 0.866), a significant TIME effect (F = 19.6, *p* < 0.001; 40–60%: *p* = 0.003, d = 0.7 moderate; 60–80%: *p* ≤ 0.001, d = 0.8 large; 80–100%: *p* < 0.001, 1.0 large), but no significant GROUP × TIME interaction (F = 0.6, *p* = 0.678).

### 3.2. Recovery

**Figure 2:**
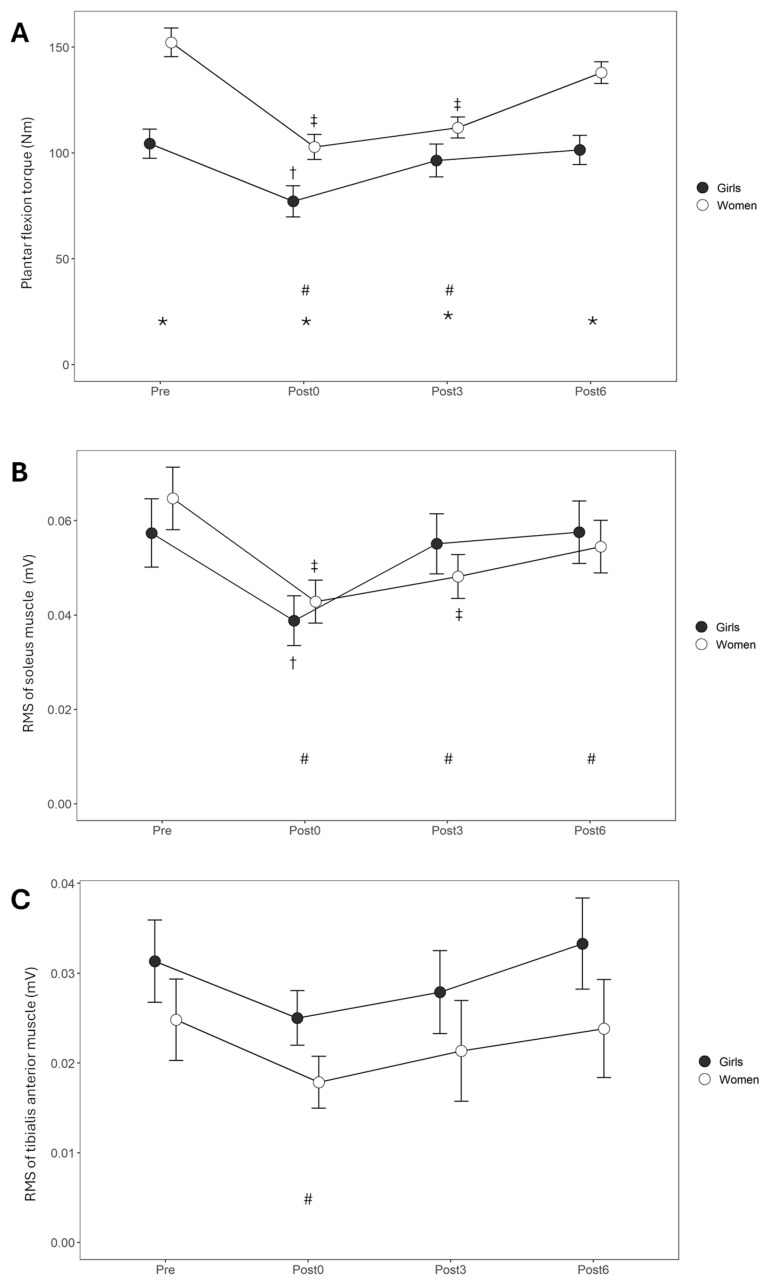
Group means and SDs of torque (**A**) and RMS EMG activity of the soleus (**B**) and tibialis anterior (**C**) muscles before (Pre) and at 0, 3, and 6 min after the fatigue protocol (Post0, Post3, and Post6, respectively). *: Significant group differences, #: Significant time effect compared to Pre, †: Significant differences for girls compared to Pre, ‡: Significant differences for women compared to Pre.

For the MVC measurements before and after fatigue (Figure 2A), torque was statistically significantly higher in women compared to girls (F = 14.6, *p* < 0.001). The effect of the TIME factor was also statistically significant (F = 44.2, *p* < 0.001), with the pre-fatigue values being higher than those of Post0 (*p* < 0.001, d = −1.5 large) and Post3 (*p* < 0.001, d = −1.0 large). A significant GROUP × TIME interaction was observed (F = 7.5, *p* < 0.001), and the post hoc test revealed that the girls demonstrated significantly lower torque at Post0 compared to the Pre values (*p* < 0.001, d = −2.2 large), whereas women exhibited significantly lower torque during Post0 (*p* < 0.001, d = −1.6 large) and Post3 (*p* < 0.001, d = −1.9 large) compared to Pre.

SOL_RMS_ before and after the fatigue protocol (Figure 2B) demonstrated no statistically significant group effect (F < 0.1, *p* = 0.966), whereas a significant time effect was observed (F = 27.4, *p* < 0.001). There were significant differences between Pre values and all measurements during recovery (Post0: *p* < 0.001, d = −0.9 large; Post3: *p* < 0.0001, d = −0.6 moderate; Post6: *p* = 0.040, d = −0.4, small). There a was significant GROUP × TIME interaction (F = 3.9, *p* = 0.012), with girls showing a significant decrease in SOL_RMS_ immediately at Post0 (*p* = 0.001, d = −1.5 large), but for women, this was the case at Post0 (*p* < 0.001, d = −1.0 large) and Post3 (*p* = 0.003, d = −0.8 large).

Regarding TA_RMS_ (Figure 2C), there was no significant effect for the GROUP factor (F = 1.6, *p* = 0.204), but the effect of TIME was statistically significant (F = 3.6, *p* = 0.017). There were significant differences observed at Post0 (*p* < 0.05, d = −0.4, moderate) compared to Pre. The GROUP × TIME interaction was not statistically significant (F = 0.2, *p* = 0.926).

**Figure 3:**
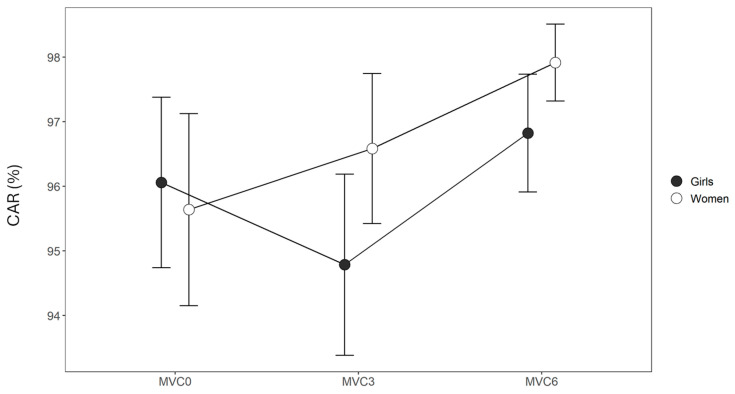
Group means and SDs of central activation ratio (CAR) during a maximal voluntary contraction (MVC) at 0, 3, and 6 min after the fatigue protocol (MVC0, MVC3, and MVC6, respectively).

As shown in Figure 3, the CAR demonstrated no significant effect for the GROUP (F = 0.4, *p* = 0.539) and TIME (F = 2.1, *p* = 0.133) factors or their interaction (F = 0.8, *p* = 0.463).

## 4. Discussion

Based on the findings of this study, the submaximal sustained isometric plantar flexion protocol resulted in an increase in the activation of the agonist and antagonist muscles (SOL_RMS_ and TA_RMS_, respectively) to a similar extent in both young and adult female handball players. On the other hand, during recovery, prepubertal females recovered faster than adults in terms of torque and agonist EMG activity. Furthermore, no differences between the groups were observed in the torque predicted by agonist muscle activity during sustained isometric contraction or in the CAR when performing MVCs during recovery time.

Findings from previous studies support the idea that the neuromuscular responses during sustained voluntary contractions are influenced by the intensity of the contraction [41,42,43]. In agreement with previous studies [22], we observed a gradual increase in EMG activity in both the agonist and antagonist muscles during a sustained submaximal isometric contraction. This increase in both muscles is supported by the concept of “common drive”, which is described as the common neural input originating from supraspinal centers and directed to different muscles during voluntary contractions [44]. The observed increase in the agonist muscle EMG could be attributed to the recruitment of additional motor units and/or an increase in their firing rate because the already recruited motor units are not capable of producing the required torque or because the excitability of these motor units is decreased [45]. In other words, in order to keep the force output or the firing rate of a motor unit constant, a stronger excitatory input is necessary when fatigue develops during a submaximal sustained contraction [46]. This regulation of motor unit recruitment might be assisted by the feedback from type III/IV afferent fibers, which are responsive to chemical stimuli from the muscle [47]. Experiments in cats have shown that type III/IV afferents remain active, even during low-intensity exercise, possibly due to mechanical rather than biochemical stimuli, since the buildup of metabolic byproducts or oxygen restriction is minimal [47]. Recent studies on humans further indicate that although these afferents provide an inhibitory input at the spinal level, their effect on the motor cortex might be facilitatory in non-fatigued conditions but inhibitory during prolonged or challenging contractions [48]. Therefore, in the present study, these afferents could play a role in the inhibition of some motor units, thereby increasing the requirement for other additional motor units that have a higher recruitment threshold and result in a greater EMG amplitude. Along these lines, high-density EMG can provide valuable insights into the recruitment patterns of motor units when fatigue develops.

Regarding fatigability in children and adults, there is a consensus that children are more fatigue-resistant to both high-intensity dynamic [49,50,51] and isometric [24,52,53] muscle contractions compared to adults. This is often attributed to factors that include children’s reduced reliance on glycolytic metabolism during intense exercise and their lower lactate production [54]. Additionally, lower muscle mass in children results in reduced intramuscular pressure and vascular occlusion, which may limit the restriction of energy substrate replenishment and the removal of metabolic by-products [55]. However, when the intensity of the fatigue-inducing contraction is lower, smaller differences between age groups are expected [22,32]. This might explain the absence of differences in the sustained isometric contraction between the age groups that was observed in the present study. One of the reasons for this result could be the fact that during low-intensity contractions, the main mechanism for energy production is based on the aerobic system, which is well developed in children and may differ minimally from that of mature adults [56]. Moreover, according to the size principle, slow-twitch motor units are recruited at low-intensity contractions, while progressively faster motor units are recruited at higher intensities [57]. This implies that differences in fatigability are more likely when muscle fiber types with different tolerance to fatigue are recruited. Considering the evidence that the proportion of fast-twitch motor units is lower in children [58,59], and that such differences in muscle fiber type distribution are more crucial at higher recruitment thresholds [60], children may show less performance decline by recruiting fewer fast-twitch motor units. Interestingly, the differences between the age groups were also absent when the EMG activity of the soleus muscle was converted to the predicted produced torque, based on the torque/EMG relationship before the sustained isometric protocol. This indirectly indicates that the amount of increase in agonist EMG due to fatigability may have similar effects on the torque output in both age groups, which implies similar fatigability at the peripheral (muscle) level.

Regarding the contributing factors to fatigability, there is a consensus that central nervous system mechanisms are primarily involved at low intensities, and as the intensity increases, peripheral factors play a more dominant role [41,42,43]. In the current study, we assessed the CAR after fatigue as an index of the level of voluntary activation, and no differences were observed between age groups or between measurements (before and 3 or 6 min after fatigue). This suggests that central factors may not play a significant role in fatigability under the conditions of the current experiment; however, it is important to underline the limitations of this method, which overestimates voluntary activation [61] and therefore is less sensitive to changes due to fatigue.

In addition to the age-related comparisons, this investigation, which assessed female handball players, has some further implications regarding how sex and training level might have influenced these findings. Firstly, it seems that the female participants of this study exhibit similar behavior to their male counterparts in studies with similar designs [32,62]. Differences in fatigability between male and female participants have been reported in the past, but it seems that when the capacity of force production is considered, differences in fatigability between sexes are diminished [63]. This implies that the level of maximal force produced by each participant might be a more important factor than sex in predicting outcomes. Considering the findings mentioned above, trained females are more likely to be able to produce higher force levels; therefore, a similar behavior to untrained men is expected regarding their fatiguability because untrained men might have similar force levels to trained females due to differences determined by their sex.

This study has several limitations that should be acknowledged. The relatively small sample size may reduce the robustness and reliability of the findings. Additionally, the use of a non-probabilistic sampling method may limit the generalizability of the results. Future research with larger, randomly selected samples is recommended to validate these findings. Furthermore, the menstrual cycle phase of female participants could not be controlled due to practical reasons (availability of participants and irregular menstrual cycles). As hormonal fluctuations across the cycle—particularly during the early follicular phase—may influence exercise performance [64], this factor might be a source of variability in our results. Future studies should consider monitoring and controlling for menstrual cycle phase in female participants to better account for potential hormonal influences on exercise performance and reduce variability in outcomes during fatiguing protocols. Additionally, participants’ nutritional intake was not monitored, which could have influenced their exercise performance [65]. Future studies should incorporate dietary monitoring to better account for the potential influence of nutrition on performance and fatigue in female handball players.

The results of the current study have some practical implications that could be addressed. We showed that although the endurance time during sustained submaximal isometric contraction was similar in prepubertal and adult females (approximately 1.5 min), their recovery rates differed significantly. Girls recovered fully in terms of torque and EMG before the third minute, whereas women required 6 min to reach the pre-fatigue values. From a practical point of view, these results translate to an exercise-to-rest ratio (E/R) of approximately 1:2 for girls and 1:4 for women. It is known that the E/R can be task-dependent, whereas the athletes’ profiles might also play a role [66]. According to our findings, despite a similar workload applied in both age groups (i.e., no differences in endurance time), the age-related differences in the E/R should be taken into account in trained female handball players, highlighting the need for tailored recovery strategies in training programs applied in different age groups. However, this should be considered with caution because the current experimental setup does not replicate training and competitive conditions, although it offers a controlled environment for more precise measures and conclusions. Furthermore, regarding the demands in a handball game, a previous study with elite male athletes showed that most maximal-intensity sprints and runs are separated by intervals of more than 1.5 min [1]. Considering this study and the present one, although we must acknowledge that there were substantial differences in the intensity (maximal vs. submaximal), duration (a few seconds vs. ~1.5 min), and type of contraction (dynamic vs. isometric), the recovery intervals for prepubertal and adult females were longer and different (3 and 6 min, respectively). This might give an advantage to younger female handball players, compared to their older counterparts, regarding fatigability, provided that children and adults have similar rest periods between activities during a handball game.

## 5. Conclusions

In conclusion, this study provides novel, age-specific insights into neuromuscular fatigue and recovery patterns in prepubertal vs. adult female handball players, focusing specifically on the soleus (SOL) and tibialis anterior (TA) muscles during a submaximal isometric fatigue protocol. The findings of this study support the notion that prepubertal and adult female handball athletes exhibit similar neuromuscular behaviors during a sustained submaximal isometric plantar flexion. Moreover, prepubertal females recover faster than adults, which has practical implications for training and is also confirmed by the relevant literature in boys and men. Further investigation into other physiological variables that change during development is required to better understand how handball training (or training in general) and sex affect fatigability. Such variables might include changes in muscle mass and hormonal fluctuations, especially during growth spurts. Finally, longitudinal data on neuromuscular profiles can guide the development of personalized recovery protocols that adapt to these changing needs, helping athletes stay injury-free and achieve peak performance in the long term.

## Data Availability

The data presented in this study are available on request from the corresponding author. The data are not publicly available due to ethical restrictions.
